# Role of the RNA-binding protein ZC3H41 in the regulation of ribosomal protein messenger RNAs in trypanosomes

**DOI:** 10.1186/s13071-023-05728-x

**Published:** 2023-03-31

**Authors:** Gloria Ceballos-Pérez, Miriam Rico-Jiménez, Claudia Gómez-Liñán, Antonio M. Estévez

**Affiliations:** 1 Instituto de Parasitología y Biomedicina “López-Neyra” (IPBLN), CSIC, Parque Tecnológico de Ciencias de la Salud, Avenida del Conocimiento, 17, 18016 Armilla, Granada Spain; 2 Present Address: Estación Experimental del Zaidín (EEZ), CSIC, Prof. Albareda 1, 18008 Granada, Spain

**Keywords:** RNA-binding proteins, *Trypanosoma brucei*, Ribosomal proteins, 5S ribosomal RNA, Zinc finger proteins, RNA immunoprecipitation sequencing

## Abstract

**Background:**

Trypanosomes are single-celled eukaryotes that rely heavily on post-transcriptional mechanisms to regulate gene expression. RNA-binding proteins play essential roles in regulating the fate, abundance and translation of messenger RNAs (mRNAs). Among these, zinc finger proteins of the cysteine3histidine (CCCH) class have been shown to be key players in cellular processes as diverse as differentiation, regulation of the cell cycle and translation. ZC3H41 is an essential zinc finger protein that has been described as a component of spliced leader RNA granules and nutritional stress granules, but its role in RNA metabolism is unknown.

**Methods:**

Cell cycle analysis in ZC3H41- and Z41AP-depleted cells was carried out using 4′,6-diamidino-2-phenylindole staining, microscopic examination and flow cytometry. The identification of ZC3H41 protein partners was done using tandem affinity purification and mass spectrometry. Next-generation sequencing was used to evaluate the effect of ZC3H41 depletion on the transcriptome of procyclic *Trypanosoma brucei* cells, and also to identify the cohort of mRNAs associated with the ZC3H41/Z41AP complex. Levels of 5S ribosomal RNA (rRNA) species in ZC3H41- and Z41AP-depleted cells were assessed by quantitative reverse transcription-polymerase chain reaction. Surface sensing of translation assays were used to monitor global translation.

**Results:**

We showed that depletion of the zinc finger protein ZC3H41 resulted in marked cell cycle defects and abnormal cell morphologies. ZC3H41 was found associated with an essential protein, which we named Z41AP, forming a stable heterodimer, and also with proteins of the poly(A)-binding protein 1 complex. The identification of mRNAs associated with the ZC3H41/Z41AP complex revealed that it is primarily composed of ribosomal protein mRNAs, and that binding to target transcripts is diminished upon nutritional stress. In addition, we observed that mRNAs encoding several proteins involved in the maturation of 5S rRNA are also associated with the ZC3H41/Z41AP complex. Finally, we showed that depletion of either ZC3H41 or Z41AP led to the accumulation of 5S rRNA precursors and a decrease of protein translation.

**Conclusions:**

We propose that ZC3H41 and Z41AP play important roles in controlling the fate of ribosomal components in response to environmental cues.

**Graphical Abstract:**

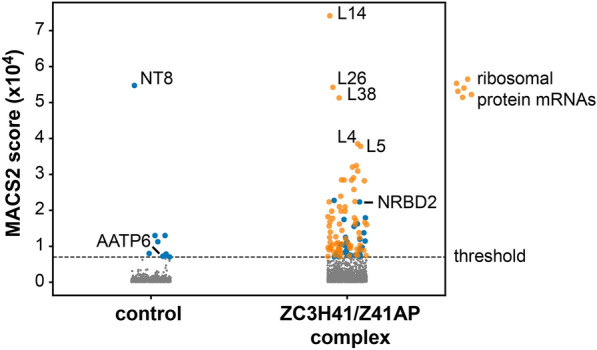

**Supplementary Information:**

The online version contains supplementary material available at 10.1186/s13071-023-05728-x.

## Background

RNA transcription, processing, modification, translation and turnover constitute a central part of cell physiology [1]. RNA metabolism often deviates dramatically from eukaryotic paradigms in trypanosomatid protozoa [2]. In these early branch organisms, RNA polymerase II transcription is polycistronic and seems to be unregulated. Individual messenger RNAs (mRNAs) are generated by coupled trans-splicing of a spliced leader (SL) RNA at the 5′-end, and polyadenylation at the 3′-end [3], leaving little room for regulation in a classical transcriptional manner. Instead, regulation of gene expression is exerted post-transcriptionally at the levels of mRNA processing, transport, mRNA turnover and translation [4]. Processing of ribosomal RNAs (rRNAs) is also unusual in trypanosomatids, as the large subunit rRNA is cleaved at several sites to yield seven stable RNA fragments [5]. Large and small subunit rRNAs, as well as 5.8S rRNA, are transcribed by RNA polymerase I, whereas RNA polymerase III transcribes 5S rRNA. Processing of RNA polymerase I-derived rRNA species requires the RNA exosome [6], the RNA helicases MTR4 and Hel66 [7, 8], and small nucleolar RNAs [9] among other factors. Proteins involved in 5S processing, on the other hand, include nuclear RNA binding domain 1 (NRBD1) and NRBD2 (p34 and p37, respectively) [10], ribosome biogenesis regulator 1 homolog (RRS1) [11], the ribosomal proteins L5 and L11 [12, 13], and a telomerase subunit La protein [14].

The family Trypanosomatidae includes many parasitic species, which are responsible for important diseases in impoverished countries around the world [15]. *Trypanosoma brucei* is the organism of choice for functional analyses in trypanosomatids, and is responsible for human and animal trypanosomiasis in sub-Saharan Africa. Trypanosomes alternate between insect (tsetse fly) and mammalian hosts, including humans, and undergo profound changes in their transcriptomes and proteomes in order to adapt to the contrasting environments they face in their life cycle [16]. Trypanosome cells proliferate as procyclic forms in the insect midgut, and migrate to the salivary glands to differentiate into non-dividing metacyclic forms, which are then transmitted through fly bites into the mammalian bloodstream. Metacyclic trypanosomes differentiate first into proliferative bloodstream forms, which in turn develop into quiescent stumpy forms that are transferred to the tsetse fly and differentiate to procyclic forms, thus completing the cycle [17].

As expected for organisms with little transcriptional control, RNA-binding proteins are of paramount importance in controlling the final levels of mRNAs and proteins [18, 19]. Among these, zinc finger proteins of the cysteine3histidine (CCCH) family have been shown to play important roles in many aspects of mRNA metabolism [19, 20]. There are 48 CCCH zinc finger proteins encoded in the genome of *T. brucei*, most of which do not seem to have homologs in other eukaryotes [20]; some of them have been studied in some detail. For example, ZFP1, ZFP2 and ZC3H18 have been shown to be involved in the differentiation of the parasite [21–23], while other zinc finger proteins, such as ZC3H5, ZC3H11, ZC3H20, ZC3H22, ZC3H32 and ZFP3 [24–29], seem to regulate mRNA turnover and translation.

ZC3H41 is an intriguing essential zinc finger protein that was detected through SL-RNA affinity purification [30], and is a component of both SL RNA granules and nutritional stress granules [30, 31]. In a previous study, we found that ZC3H41 bound non-specifically to a purine-responsive RNA regulatory element, probably as a consequence of the interaction with the SL bridging sequence used for RNA chromatography [32]. Moreover, ZC3H41 seems to accumulate in vesicles that are released into the extracellular medium [30]. In order to gain insight into the role of this protein in RNA metabolism, we analyzed the transcriptome of ZC3H41-depleted trypanosomes and also the subset of mRNAs associated with this protein. We found that ribosomal protein mRNAs, as well as transcripts encoding several factors involved in the maturation of 5S rRNA, are particularly enriched in the ZC3H41-bound transcriptome. We also show that ZC3H41 plays a role in the maturation of 5S rRNA, and that its depletion results in a decrease of global translation.

## Methods

### Trypanosome culture

*Trypanosoma brucei* 449 procyclic cells [33] were grown at 28 ºC in SDM-79 medium [34] containing 10% fetal bovine serum. For starvation experiments, cells were collected from logarithmic cultures, washed twice in phosphate-buffered saline (PBS), resuspended in the original volume of PBS and incubated at 28 ºC for 3 h with gentle shaking. Transgenic trypanosomes were obtained following standard procedures [35].

### Expression of tandem affinity purification fusion proteins and immunoblot

For expression of N-terminal tandem affinity purification (TAP) tagged proteins, fragments of ZC3H41 (Tb927.11.1980) or Z41AP (Tb927.7.7460) open reading frames (ORFs) were cloned into p2676 [36] to yield pGR307 and pGR315, respectively. These plasmids were transfected into procyclic 449 cells and selected in the presence of 1 µg/ml puromycin. Oligonucleotides used for cloning are described in Additional file 1. Detection of TAP-tagged proteins was carried out by immunoblot analysis using Papanicolaou reagent (Sigma) or an anti-protein A antibody (Sigma), whereas 4xTy-tagged protein levels were monitored using a BB2 monoclonal antiserum [37]. Other antisera used were anti-α-tubulin (clone B-5-1-2; Sigma), anti-p34/p37 (NRBD1/2; [38]), anti-P0 [39], anti-S9 [unpublished; a kind gift from Christine Clayton (Zentrum für Molekulare Biologie der Universität Heidelberg, Heidelberg)], anti-DRBD3 [40], anti-RRP4 [6] and anti-puromycin (clone 12D10; Sigma). To prepare total cell extracts for immunoblot analysis, cells were harvested from logarithmic cultures, lysed in Laemmli buffer, boiled, and loaded directly in sodium dodecyl sulfate-polyacrylamide gel electrophoresis gels (1–2 × 10^6^ cells/lane). Proteins were visualized using either chemiluminescence or an Odyssey CLx Near-Infrared Fluorescence Imaging System (LI-COR Biosciences).

### RNA interference

For RNA interference (RNAi) studies, a stem-loop strategy was followed to generate double-stranded RNA [35]. A fragment of the *ZC3H41* gene corresponding to the last 398 base pairs (bp) of the ORF plus 101 bp of the 3’-untranslated region, or a fragment of the *Z41AP* gene corresponding to the last 381 bp of the ORF plus 16 bp of the 3’-untranslated region, were amplified and cloned into pGR19 [35] to yield pGR309 and pGR318, respectively. Additionally, an alternative RNAi plasmid was generated for ZC3H41 using a fragment corresponding to positions 736–1101 of the ORF to yield pGR440. Trypanosomes expressing TAP-ZC3H41 or TAP-Z41AP were transfected with NotI-linearized pGR309, pGR318 or pGR440 and selected in the presence of 50 µg/ml hygromycin. For RNAi induction, tetracycline was added to the culture medium at a concentration of 1 µg/ml.

### Immunofluorescence and cell cycle analysis

Immunolocalization studies were carried out as previously described [40] using an anti-protein A antiserum (Sigma). For cell cycle analysis using 4′,6-diamidino-2-phenylindole (DAPI) staining and microscopic examination, uninduced or ZC3H41-depleted cells were counted on slides of fixed trypanosomes labeled with DAPI. To aid the visualization of cells, trypanosomes were incubated with an anti-EP1 monoclonal antibody (Cedarlane). At least 500 cells were counted for each time point. For cell cycle analysis using flow cytometry, 2.5 × 10^7^ trypanosomes from uninduced or RNAi-induced cultures were collected by centrifugation at 1400* g* for 10 min at 4 °C and washed once in cold PBS. Cell pellets were fixed overnight at – 20 ℃ in 70% cold ethanol, washed once in cold PBS, resuspended in 0.5 ml PBS containing 40 μg/ml propidium iodide (Sigma) and 10 μg/ml RNase A (Thermo Scientific), and incubated for 30 min at room temperature. The DNA content of propidium iodide-stained cells was measured with either a FACSCalibur or a FACSymphony flow cytometer (Becton Dickinson) using a FL3 detector. The percentages of cells in subG1, G1/S and G2/M phases, as well as those having more than two nuclei (> 2N), were determined using the FlowJo software. For each time point, 20,000 events per sample were measured. Gates were set manually using uninduced cells, and the same settings were applied to all samples.

### TAP and mass spectrometry

Proteins complexes were purified from 1–2 × 10^10^ cells using the TAP method [41] with the modifications described in [42]. After SYPRO staining, individual bands were excised, subjected to matrix-assisted laser desorption/ionization–time-of-flight/time-of-flight analysis in a UltrafleXtreme mass spectrometer (Bruker). Protein identification was assigned by peptide mass fingerprinting and confirmed by MS/MS analysis of at least two peptides in each sample. Mascot 2.0 search engine (Matrix Science) was used for protein identification. RNase A + T1 treatment was carried out as in [42].

### Ultraviolet crosslinking and polynucleotide kinase assays

The method described in [43] was followed with some modifications, whereby 2 × 10^9^ trypanosome cells expressing TAP fusion proteins were washed in serum-free SDM-79 medium, and resuspended in 2 ml of ice-cold Voorheis’s-modified PBS (PBS supplemented with 10 mM glucose and 46 mM sucrose). Half of the cells were ultraviolet (UV) irradiated twice at 400 mJ/cm^2^ in a Stratalinker apparatus (Stratagene) in a single well of a 6-well culture plate on ice. Cell suspensions were centrifuged, snap frozen in liquid nitrogen and stored at – 80 ºC until use. Pellets were resuspended in 0.5 ml of 10 mM Tris–HCl, pH 7.4, 2 mM dithiothreitol (DTT), 5 mM MgCl_2_, 0.5% IGEPAL CA-630, and protease inhibitors (complete mini ethylenediaminetetraacetic acid-free cocktail; Roche) and lysed by brief vortexing. Cell extracts were centrifuged at 16,000* g* for 10 min at 4 ºC. Supernatants were treated with 100 µg of RNaseA (Sigma) and 10 units of DNase I (Promega) for 30 min on ice; NaCl was added at a final concentration of 150 mM, and the mixture was incubated for 2 h at 4 ºC in the presence of 1 mg of paramagnetic epoxy beads (M-270; Invitrogen) coupled with rabbit immunoglobulin G (Sigma) according to the manufacturer’s instructions. Beads were washed four times with 10 mM Tris–HCl, pH 7.4, 1 M NaCl, 0.5% IGEPAL and twice with T4 polynucleotide kinase (PNK; Promega) buffer (40 mM Tris–HCl, pH 7.5, 50 mM NaCl, 10 mM MgCl_2_, 5 mM DTT, 0.1% IGEPAL CA-630), resuspended in 20 µl PNK buffer containing 30 µCi [γ-^32^P]ATP (6000 Ci/mmol; Perkin Elmer) and 10 units of PNK (Promega), incubated for 20 min at 37 ºC in a Thermomixer at 800 r.p.m., washed thrice in PNK buffer lacking DTT, and subjected to sodium dodecyl sulfate-polyacrylamide gel electrophoresis for PhosphorImager analysis and immunoblot.

### High-throughput sequencing and bioinformatics

For transcriptome analyses of ZC3H41-depleted trypanosomes, RNAi was induced for 2 days, and total RNA was obtained using the RNeasy Mini Kit (Qiagen). mRNA libraries were obtained using the TruSeq Stranded mRNA sample preparation protocol (Illumina). Biological triplicates were processed in parallel with a high-throughput sequencing (RNA-seq) of control procyclic 449 cells described in [44] [Gene Expression Omnibus (National Center for Biotechnology Information; NCBI) series GSE186570; samples GSM5655679, GSM5655680 and GSM5655681] and sequenced at the Genomics Unit of the Instituto de Parasitología y Biomedicina “López-Neyra”–Consejo Superior de Investigaciones Científicas (Granada, Spain) using a NextSeq 500 platform (Illumina). To identify transcripts associated with ZC3H41/Z41AP complexes (RNA immunoprecipitation sequencing; RIP-seq), two independent purifications from cell lines expressing TAP-Z41AP were processed in parallel with a RIP-seq of the PuREBP1/2 complex described in [32] [Gene Expression Omnibus (NCBI) series GSE145466; samples GSM4318666, GSM4318667, GSM4318669 and GSM4318671].

The resulting 76-nucleotide paired-end sequences were checked for quality using FastQC (https://www.bioinformatics.babraham.ac.uk/projects/fastqc/) and mapped to the *T. brucei* 11 megabase chromosomes (TREU927, version 5.7) using the align module of the Subread package (Rsubread version 1.34.7; [45]) with the options nTrim5 = 4, nTrim3 = 1, unique = TRUE. Pearson correlation coefficients between replicates were > 0.980 in all cases.

For differential expression analysis, reads assignment to mRNAs and counting were carried out using the featureCounts program of the Subread package (version 1.5.0-p [46]) with parameters –p –B –C –ignoreDup –Q 20. Counts were analyzed using edgeR (version 3.36.0; [47]); only genes containing more than one count per million mapped reads in at least three samples were considered. For Pearson correlation analysis, counts per million mapped reads were normalized using the trimmed means of the *M* values method [47] and transformed to log_2_ in edgeR. For coverage plots, regions of interest were binned and counted (sliding window, 100 bp; step size, 10 bp) using the countReadsPerBin module of deepTools API (version 3.5.0; [48]), and corrected for library size.

RIP-seq data were analyzed using either edgeR as above, comparing input RNA with immunoprecipitated RNA samples, or peak calling with MACS2 (version 2.2.7.1, [49]). In the latter case, peak calling was performed using the module callpeak on the binary alignment map (BAM) files obtained after alignment to the reference genome; the options were –q 0.005 –g 3.5e7 —nomodel –f BAMPE. BAM files corresponding to input RNA were used as controls for the peak calling. The resulting narrowPeak files obtained from both biological duplicates were intersected using the intersect module of bedtools [50] with options –f 0.9 –r. The final peak list was further intersected with a browser extensible data file containing the coordinates of all genes (ORFs plus untranslated regions) present in the *T. brucei* 11 megabase chromosomes (TREU927; version 5.7) using option –f 0.5.

Gene ontology analysis was performed in TriTrypDB [51] using gene ontology slim terms; evidence was set to both computed and curated.

### Quantitative reverse transcription–polymerase chain reaction

PCR conditions, calculations and small-scale RNA immunoprecipitations were done as described [32] using *AATP11* mRNA (Tb927.4.4730) as a reference. All quantitative reverse transcription–polymerase chain reaction (RT-PCR) experiments were performed with at least three biological replicates. Oligodeoxynucleotide pairs used are listed in Additional file 1.

### Translation assay

The surface sensing of translation assay [52] was used to monitor global translation according to a procedure described for trypanosomes [8]. Briefly, 1 × 10^7^ trypanosomes were deposited in 24-well plates, incubated in the presence of 10 µg/ml puromycin for 30 min at 28 ºC, washed in serum-free medium at the same temperature and subjected to immunoblot analysis as described above. Two controls were included: cells not treated with puromycin, and cells incubated for 30 min with 50 µg/ml of cycloheximide prior to the addition of puromycin. Puromycinilated peptides were detected with an anti-puromycin antibody (clone 12D10, 1:5000; Sigma) in an Odyssey Imaging System, and quantified using Image Studio (version 5.2; LI-COR Biosciences). DRBD3, which is stable over the course of the experiment [53], was used for normalization.

## Results

### Depletion of ZC3H41 results in severe cell cycle defects

**Fig. 1a–e:**
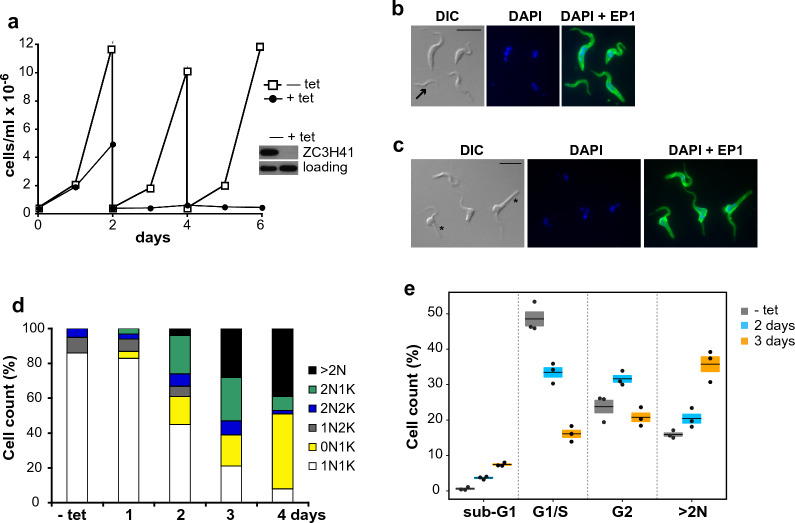
Depletion of ZC3H41 leads to abnormal cell morphologies and defects in cell division. **a** Effect of ZC3H41 depletion on cell growth. Cells expressing tandem affinity purification (TAP) tag-ZC3H41 were transfected with a plasmid expressing double-stranded RNA (dsRNA) corresponding to ZC3H41 in a tetracycline (tet)-inducible fashion. Cell cultures were followed for up 6 days and diluted every 2 days as needed. Depletion of TAP-ZC3H41 was confirmed by western blot after 2 days of tet induction (inset); RRP4 was included to assess equal loading. **b**,** c** Analysis of cell morphology in ZC3H41-depleted cells that were RNA interference (RNAi)-induced for 2 (**b**) or 3 (**c**) days with tet. Cells were stained with an anti-EP1 antibody to aid in their visualization, and mounted in mounting medium containing diamidino-2-phenylindole (DAPI). Bar represents 10 µm. A zoid is indicated with an arrow in **b**; multinucleated cells exhibiting the ‘nozzle phenotype’ are indicated with asterisks in **c**. **d** Nucleus (N) and kinetoplast (K) configurations of individual cells were analyzed by DAPI staining. The percentage of cells with physiological (1N1K, 1N2K and 2N2K) or aberrant karyotypes (0N1K, 2N1K and > 2N) is shown for uninduced (− tet) or ZC3H41-depleted cells induced for 1, 2, 3 or 4 days with tet. **e** Cell cycle analysis of uninduced (− tet) or ZC3H41-depleted cells induced for 2 or 3 days with tet. Percentages of cells in sub-G1, G1/S or G2 phases, as well as those having more than two nuclei (> 2N), are expressed as the mean (horizontal lines) ± SEM (shaded areas) of three independent RNAi inductions (dots)

To gain more insight into the function of ZC3H41, depletion in vivo using RNAi was used. We first generated a trypanosome procyclic cell line that expressed a TAP-tagged version from the endogenous locus. TAP-ZC3H41 was detected as a single band of the expected size in western blot analysis of total cell extracts (Additional file 2). Endogenous ZC3H41 is mainly a cytosolic protein [30, 54] and TAP-ZC3H41 localized also in the cytosol, as judged by immunofluorescence assays using antibodies against the TAP tag (Additional file 2). Trypanosomes expressing TAP-tagged ZC3H41 were further transfected with a vector expressing double-stranded RNA against ZC3H41 in a tetracycline-inducible manner. TAP-ZC3H41 was efficiently depleted upon tetracycline induction, and a marked reduction in cell growth was observed (Fig. 1a), indicating that ZC3H41 is essential in procyclic trypanosomes, in agreement with previous results [30].

ZC3H41-depleted cells were examined for morphological and karyotypic defects using DAPI staining and microscopic examination. We could detect small cells corresponding to anucleate parasites after 1–2 days of RNAi induction (0N1K or zoids; Fig. 1b). Cells exhibiting an elongated end (nozzle phenotype; [21]) were observed after 3 days of ZC3H41 ablation (Fig. 1c). A time-dependent reduction in the proportion of cells containing one nucleus and one kinetoplast (1N1K; which are in the G1 or S phases of the cell cycle [55]), and a concomitant increase in zoids and multinucleated cells (> 2N) was readily observed; after 2 days of RNAi induction, the majority of cells had aberrant karyotypes (Fig. 1d). Both the decrease in 1N1K cells and the increase in > 2N species were confirmed by flow cytometry (Fig. 1e; representative flow cytometry plots can be seen in Additional file 2). Inhibition of trypanosome growth and appearance of zoids and ‘nozzled cells’ were also observed when a different, non-overlapping gene fragment was used for RNAi-mediated ablation of ZC3H41 (Additional file 3), which ruled out off-target effects.

### ZC3H41 forms a heterodimer with Tb927.7.7460

**Fig. 2a–e:**
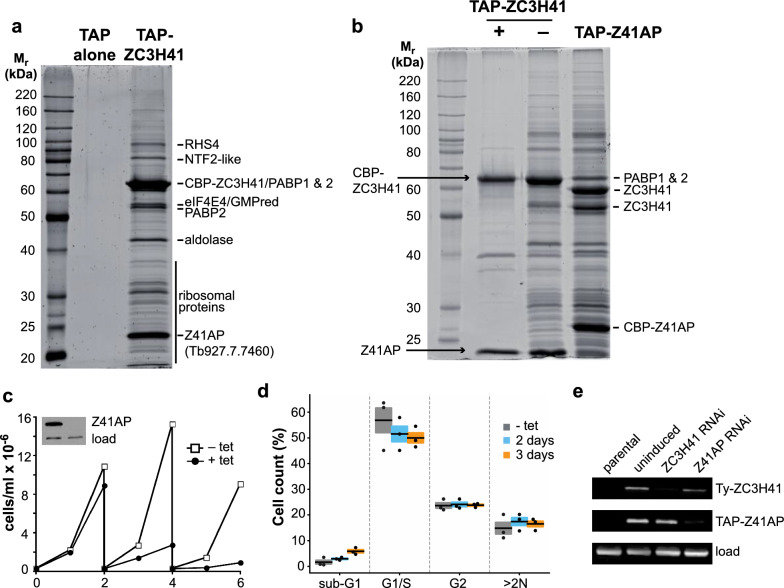
ZC3H41 is found associated with Tb927.7.7460 (Z41AP) in a stable complex. **a**,** b** Sodium dodecyl sulfate-polyacrylamide gel electrophoresis/SYPRO staining of TAPs using TAP alone, TAP-ZC3H41 or TAP-Z41AP proteins as baits. The calmodulin binding peptide (CBP) tag remains after tobacco-etch virus (TEV) protease cleavage in the TAP procedure. Proteins identified by mass spectrometry are indicated (a complete list is presented in Additional file 4: Table S2). TAP-ZC3H41 purifications carried out in the absence (−) or presence (+) of RNases A and T1 are shown in **b**. **c** Z41AP is essential in procyclic trypanosomes. A cell line expressing TAP-Z41AP was transfected with a plasmid producing dsRNA corresponding to Z41AP in a tet-dependent manner. Cell cultures were followed for up to 6 days and diluted every 2 days as needed. Depletion of TAP-Z41AP was confirmed by immunoblot after 2 days of tet induction (inset; see also **e**); RRP4 was used to assess equal loading. **d** Cell cycle analysis of uninduced (− tet) or Z41AP-depleted cells induced for 2 or 3 days with tet. Percentages of cells in sub-G1, G1/S or G2 phases, as well as those having more than two nuclei (> 2N), are expressed as the mean (horizontal lines) ± SEM (shaded areas) of three independent RNAi inductions (dots). **e** Effect of ZC3H41 or Z41AP depletion on the levels of the corresponding protein partner. A cell line expressing both 4xTy-ZC3H41 and TAP-Z41AP was transfected with plasmids expressing dsRNA corresponding to either ZC3H41 or Z41AP. Protein levels were monitored by immunoblot using BB2 or anti-protein A antisera that detect 4xTy or TAP tags, respectively. α-tubulin was used as a loading control. For other abbreviations, see Fig. 1

We used the TAP technique to assess whether ZC3H41 binds to other proteins in the cell. ZC3H41 was found associated in apparent stoichiometry with a 22-kDa protein, Tb927.7.7460, and to a lesser extent with several other proteins (Fig. 2a; Additional file 4). Tb927.7.7460, henceforth named ZC3H41-associated protein (Z41AP), which is also mainly cytosolic [54], contains a SKP1 motif (S-phase kinase-associated protein, a component of the ubiquitin ligase complex; [51]), and was also detected upon SL-RNA affinity purification ([30]; named p22 in that study). We performed a reciprocal TAP using Z41AP as bait, and obtained a very similar protein pattern, of which ZC3H41 was the most prominent band in this case (Fig. 2b). Most of the protein interactions were lost when the cell lysate was treated with a mixture of RNases A and T1; the association of ZC3H41 with Z41AP, however, was resistant to RNases digestion (Fig. 2b). Moreover, both proteins remain associated with each other in the presence of a high salt concentration (see below), and are found at roughly equal levels within the cell according to proteomic surveys (Additional file 5; [53, 56]). These results strongly suggest that ZC3H41 and Z41AP form a stable heterodimer in vivo. Silencing of Z41AP expression by RNAi led to inhibition of cell growth (Fig. 2c), indicating that this protein is also essential in procyclic trypanosomes. In contrast to ZC3H41 RNAi, however, depletion of Z41AP resulted in minimal cell cycle defects, as judged by flow cytometry (Fig. 2d). To study whether the depletion of ZC3H41 had an effect on the levels of Z41AP and vice versa, we generated a cell line that expressed both proteins with a different tag (4xTy-ZC3H41 and TAP-Z41AP). The resulting cell line was transfected with plasmids targeting either ZC3H41 or Z41AP for RNAi. As shown in Fig. 2e, the independent depletion of either protein had no effect on the levels of the corresponding partner, indicating that both proteins are stable as free species within the cell.

### ZC3H41 and Z41AP are RNA-binding proteins

**Fig. 3a, b:**
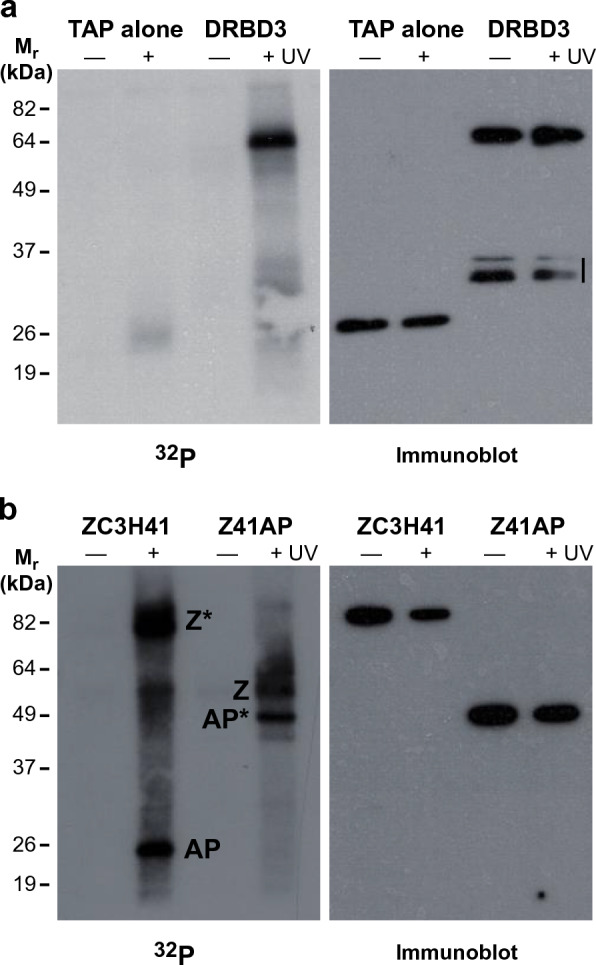
ZC3H41 and Z41AP bind RNA in vivo. Cell lines expressing TAP-DRBD3, TAP-ZC3H41, TAP-Z41AP or TAP tag alone were irradiated (+) or not (−) with ultraviolet light. TAP-tagged proteins were immunoprecipitated using paramagnetic immunoglobulin G beads, end-labeled with [γ-^32^P]ATP and polynucleotide kinase (PNK), and subjected to sodium dodecyl sulfate-polyacrylamide gel electrophoresis and PhosphorImager detection [^32^P (left panels)]; TAP-tagged proteins were also visualized by immunoblot using Papanicolaou reagent [Immunoblot (right panels)]. **a** Trypanosomes expressing the TAP tag alone or TAP-DRBD3 were used as negative or positive controls, respectively; bands below TAP-DRBD3 probably represent degradation products (vertical line). **b** Immunoprecipitation and PNK labeling of cells expressing either TAP-ZC3H41 (Z) or TAP-Z41AP (AP). TAP-tagged species are indicated with asterisks. For other abbreviations, see Figs. 1 and 2

ZC3H41 and Z41AP were purified upon SL-RNA chromatography [30], and both proteins were detected in the trypanosome mRNA-bound proteome [57], which suggests a role as RNA-binding proteins. To validate that ZC3H41 and Z41AP are able to bind RNA in vivo, living trypanosomes were irradiated with UV light, and ZC3H41/Z41AP ribonucleoprotein complexes were immunoprecipitated (from cell lines expressing either TAP-ZC3H41 or TAP-Z41AP) and washed in high-salt conditions (1 M NaCl); associated RNAs were then end-labeled using polynucleotide kinase and [γ-^32^P]ATP. Cell lines expressing the TAP tag alone or TAP-DRBD3/PTB1, a bona fide RNA-binding protein [40, 58], were used as negative or positive controls, respectively. As shown in Fig. 3, strongly labeled bands of the expected sizes could be observed in UV-treated cells, whereas no or low labeling was detected in non-irradiated samples or in the cell line expressing the TAP tag alone (Fig. 3a). These results indicate that ZC3H41 and Z41AP are genuine RNA-binding proteins.

### Depletion of ZC3H41 has minor effects on the transcriptome of procyclic trypanosomes

**Fig. 4a–d:**
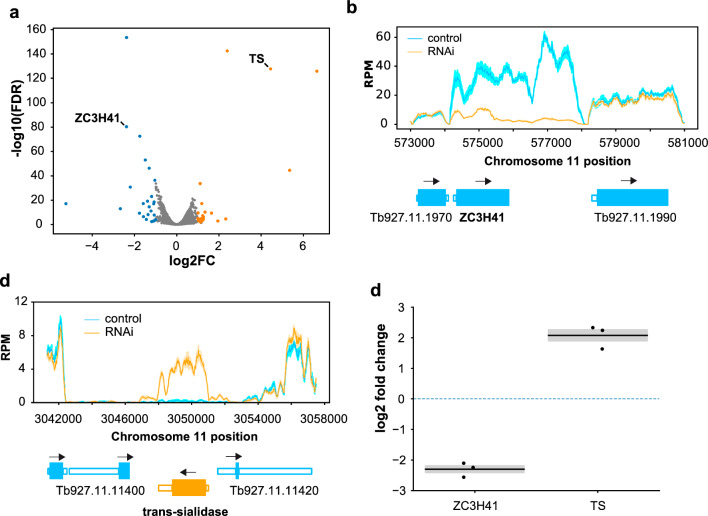
Effects of ZC3H41 depletion on the transcriptome of procyclic *Trypanosoma brucei*. **a** Volcano plot of differential gene expression in ZC3H41-depleted cells compared to control cells. Blue dots correspond to downregulated transcripts, orange dots indicate upregulated transcripts, and grey dots represent unregulated messenger RNAs (mRNAs); thresholds for differential abundance were |log2 fold change|> 1 and false discovery rate < 0.01. **b**,** c** Coverage plots of *ZC3H41* and Tb927.11.11410 loci. Read counts were analyzed with sliding windows (100-base pair bins, 10-base pair steps) and normalized by library size (reads per million). Open-reading frames are represented as thick, filled boxes, untranslated regions as thin, open boxes; arrows indicate the direction of transcription. Reads per million values are represented as the mean (solid lines) ± SEM (shaded area) of three RNA-seq biological replicates. **d** Quantitative reverse transcription–polymerase chain reaction (RT-PCR) analysis to confirm proper ablation of *ZC3H41* mRNA and upregulation of trans-sialidase (TS) transcript. Fold changes (log2 converted) are expressed as the mean (horizontal lines) ± SEM (shaded areas) of three independent RNAi inductions in procyclic trypanosomes (dots). The horizontal dashed line indicates log2 = 0, i.e. no change in gene expression

The transcriptome of ZC3H41-depleted cells was examined by high-throughput sequencing (RNA-seq) after 2 days of tetracycline induction, and compared to that of control cells. Differential abundance analyses were carried out with edgeR using cutoffs of 1 for |log2| fold change (i.e. ± twofold linear fold change) and 0.01 for false discovery rate (FDR). A volcano plot for the differentially expressed genes in ZC3H41-silenced genes is shown in Fig. 4a. Only 44 transcripts were differentially expressed, including the mRNA encoding ZC3H41, as expected (Figs. 4a, b; Additional files 6, 7). In general, ZC3H41-regulated transcripts seem to be expressed at low abundance, and almost half of them (43%) are probably non-coding, since the corresponding encoded proteins have not been detected in proteomic surveys (Additional file 6). A few mRNAs differentially regulated by ZC3H41 were previously observed to be also differentially regulated upon depletion of other proteins (Additional file 6). A transcript encoding a trans-sialidase (Tb927.11.11410) was clearly upregulated in ZC3H41-depleted cells (Fig. 4a, c; Additional files 6, 7). This transcript is poorly expressed in procyclic cells (Fig. 4c; Additional file 7; [51]). Interestingly, the corresponding gene is located in reverse orientation within a polycistronic transcriptional unit (Fig. 4c), and the encoded trans-sialidase seems to be expressed at least in bloodstream forms, as judged by mass spectrometry analysis [56, 59]. Downregulation of *ZC3H41* and upregulation of trans-sialidase Tb927.11.11410 transcripts were confirmed by quantitative RT-PCR (Fig. 4d).

### ZC3H41/Z41AP complex binds to ribosomal protein mRNAs

**Fig. 5a–c:**
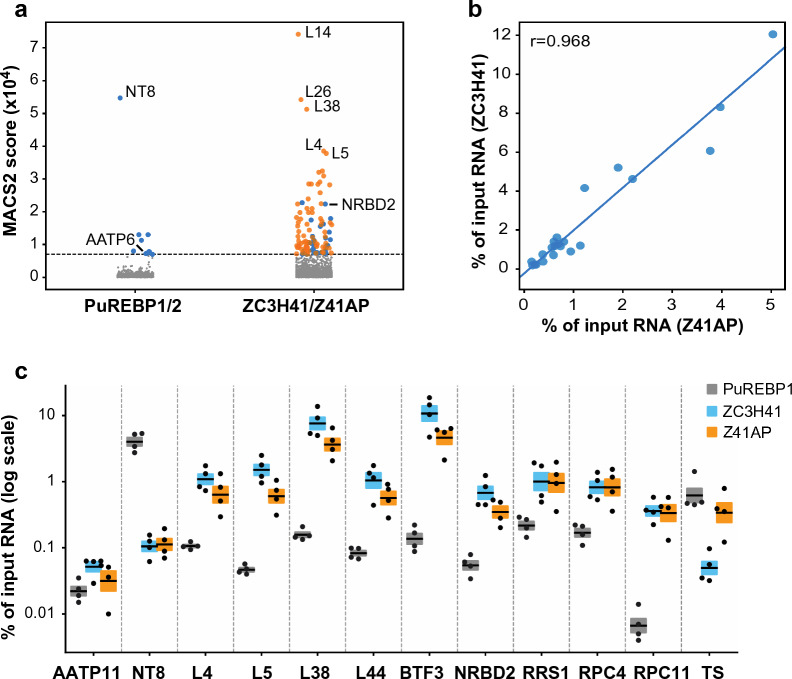
Identification of the cohort of mRNAs bound to the ZC3H41-Z41AP complex by RNA immunoprecipitation sequencing (RIP-seq). **a** MACS2 analysis of the PuREBP1/2- and ZC3H41/Z41AP-bound transcriptomes. Immunoprecipitated transcripts identified by MACS2 were ranked according to their MACS2 score. A threshold of 7000 (horizontal dashed line) was set up based on the scores of *NT8* and *AATP6*, which are bona fide targets of the PuREBP1/2 complex. Blue dots and grey dots correspond to transcripts that are, or are not, highly likely to be genuine targets, respectively. Orange dots indicate mRNAs encoding ribosomal proteins; none was detected above threshold scores in PuREBP1/2 purifications. **b** Correlation analysis of RIP followed by quantitative RT-PCR obtained using TAP-Z41AP or TAP-ZC3H41 as baits (see below); values indicate percentages of immunoprecipitated transcripts relative to input RNA. **c** RIP followed by quantitative RT-PCR to confirm association of the indicated transcripts to either ZC3H41, Z41AP or PuREBP1 (as a negative control). *AATP11* (and *NT8* in ZC3H41 and Z41AP purifications) were used as non-bound controls. *L38* data correspond to the Tb927.9.2020 paralog (see Additional File 8 for the Tb927.10.3280 paralog). Percentages of immunoprecipitated RNA relative to input RNA are expressed as the mean (horizontal lines) ± SEM (shaded areas) of four independent RIPs (dots). For other abbreviations, see Figs. 1, 2 and 4

We showed above that ZC3H41 and Z41AP are bona fide RNA-binding proteins. To identify the cohort of mRNAs bound to the ZC3H41/Z41AP complex, we performed TAP followed by high-throughput sequencing (RIP-seq) using Z41AP as bait. Z41AP-TAP purification, generation of RNA-seq libraries and Illumina sequencing were done in parallel with a RIP-seq of another RNA-binding protein complex, PuREBP1/2 [32], and thus we used the latter TAP as a control to identify mRNAs specifically associated with ZC3H41/Z41AP. The two datasets were analyzed using either edgeR or the peak caller MACS2 to identify mRNAs enriched in the TAP-purified fractions; both approaches haven been used successfully in RIP-seq experiments [32, 60–62]. MACS2 scores showed better correlation with quantitative RT-PCR data (see below) than edgeR logFC values (Pearson’s *r* = 0.559 vs 0.242; Additional file 8), and therefore we used the former approach to assess for specificity. Based on the MACS2 scores obtained for *NT8* and *AATP6* mRNAs in the PuREBP1-TAP dataset, which are known components of the PuREBP1/2 complex [32], we considered transcripts showing MACS2 score values > 7000 as highly likely to be genuine ZC3H41/Z41AP targets. This gave us a list of 104 mRNAs (Fig. 5a; Additional file 9). There was a notable abundance of mRNAs encoding ribosomal proteins, which comprised 69% of all transcripts in the final list (Fig. 5a; Additional file 9). Moreover, the top 40 scoring transcripts corresponded almost exclusively (38/40) to ribosomal protein mRNAs (Additional file 9). Accordingly, gene ontology analysis of this subset revealed a clear enrichment in ‘ribosome biogenesis’ and ‘translation’ categories within the ‘biological process’ ontology (FDR < 0.0001), and in ‘structural constituent of ribosome’ and ‘rRNA binding’ categories within the ‘molecular function’ ontology (FDR < 0.0001).

To confirm binding of target mRNAs to the ZC3H41/Z41AP complex, we performed quantitative RT-PCR assays following RIP using either TAP-ZC3H41 or TAP-Z41AP as baits; PuREBP1-TAP purifications were used as negative controls. We observed a strong correlation (Pearson’s *r* = 0.968) between TAP-ZC3H41 and TAP-Z41AP quantitative RT-PCR enrichment values (Fig. 5b), which further supports the association of both proteins within the cell and strengthens the specificity of the assay. We could confirm the association of ZC3H41/Z41AP complex with transcripts encoding ribosomal proteins L4, L5, L7a, L14, L36, L37, L44, S8 and two paralogs of L38, the basic transcription factor BTF3, the RNA-binding protein NRBD2 (also known as p37 [10], which has been described as a constituent of the ribosome [63]), the ribosomal biogenesis regulatory protein RRS1 [11], the subunits of RNA polymerase III RPC4 and RPC11, and the translation factor eIF1A (Fig. 5c; Additional file 8). All these transcripts were significantly more enriched (two-sided Student's t-test, *n* = 4, *P* < 0.05) in TAP-ZC3H41 or TAP-Z41AP purifications than in PuREBP1-TAP samples (Fig. 5c; Additional file 8). This did not represent inefficient immunoprecipitation of PuREBP1/2 ribonucleoprotein complexes since *NT8* mRNA could be readily detected upon TAP purification of PuREBP1 but not in TAP-ZC3H41 or TAP-Z41AP purifications (Fig. 5c). The transcript encoding the transialidase Tb927.11.11410 was not found significantly associated with ZC3H41/Z41AP (TS; Fig. 5c), and was not detected in MACS2 or edgeR analyses either. Therefore, the increase in transialidase mRNA abundance observed in ZC3H41-depleted cells was probably due to secondary effects unrelated directly to ZC3H41/Z41AP binding.

Using immunoblot assays, we next assessed whether the abundance of some proteins encoded by ZC3H41/Z41AP-bound transcripts was affected in ZC3H41-depleted trypanosomes. As shown in Additional file 8, neither the levels of NRBD2 nor those of ribosomal proteins P0 or S9 (whose corresponding transcripts were also found associated with ZC3H41/Z41AP; see Additional file 9) were altered after 48 h of RNAi induction.

### The association of ZC3H41/Z41AP with target mRNAs decreases under nutritional stress

**Fig. 6a, b:**
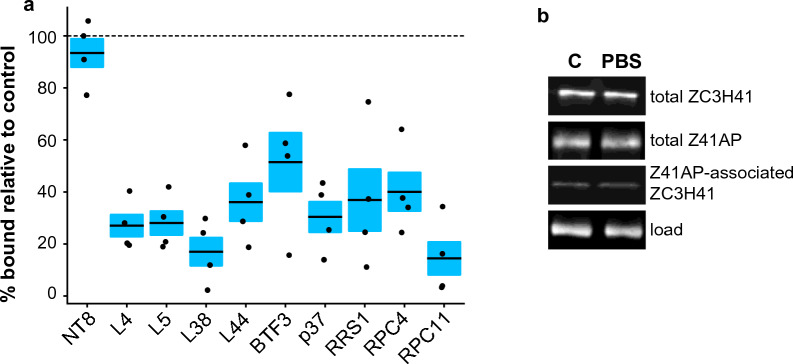
Association of ZC3H41/Z41AP with target mRNAs under nutritional stress. **a** Cells expressing TAP-ZC3H41 were incubated in normal growth medium or in phosphate-buffered saline for 3 h, and association with the indicated transcripts was assessed by RIP followed by quantitative RT-PCR. *NT8* transcript was used as a non-bound control. Percentages of immunoprecipitated RNA under starvation stress relative to those measured in normal conditions are expressed as the mean (horizontal lines) ± SEM (shaded areas) of four independent RIPs (dots). The horizontal dashed line indicates 100% binding in starvation relative to normal growth (i.e. no changes in binding). **b** ZC3H41 and Z41AP levels are unaltered in starved cells. Double-tagged cells expressing both 4xTy-ZC3H41 and TAP-Z41AP were subjected to starvation as described above. Total ZC3H41 or Z41AP levels, as well as those of ZC3H41 associated with Z41AP upon TAP purification, were assessed by immunoblot using antibodies against the tags. α-tubulin served as a loading control for total levels. For other abbreviations, see Figs. 1, 2, 4 and  5

Upon nutritional starvation, both ZC3H41 and Z41AP accumulate in stress granules, but mRNAs encoding ribosomal proteins are underrepresented in these structures [31]. Since we showed above that ZC3H41/Z41AP complex binds mainly to transcripts coding for ribosomal components in normal growth conditions, we next assessed whether there was a decrease in the association of ZC3H41/Z41AP with mRNAs encoding ribosomal proteins upon nutritional stress. Cells expressing TAP-ZC3H41 were subjected to nutritional starvation for 3 h, and binding of the ZC3H41/Z41AP complex to target transcripts was tested by RIP followed by quantitative RT-PCR as above. Indeed, we could observe a significant decrease in binding (two-sided Student’s *t*-test, *n* = 4, *P* < 0.05) in all tested ZC3H41/Z41AP-associated mRNAs upon nutritional stress (Fig. 6a). Loss of binding under starvation does not seem to be a general effect due to stress, since other trypanosomal RNA-binding proteins remain associated with their target transcripts in stress conditions [42] or are even more prone to associate with mRNAs [64].

We also assessed whether the expression of ZC3H41 or Z41AP was itself altered under nutritional stress. As shown in Fig. 6b, the levels of total ZC3H41, total Z41AP, and Z41AP-associated ZC3H41 remained unchanged under starvation.

### ZC3H41 and Z41AP are involved in 5S rRNA biogenesis, and their depletion results in decreased global translation

**Fig. 7:**
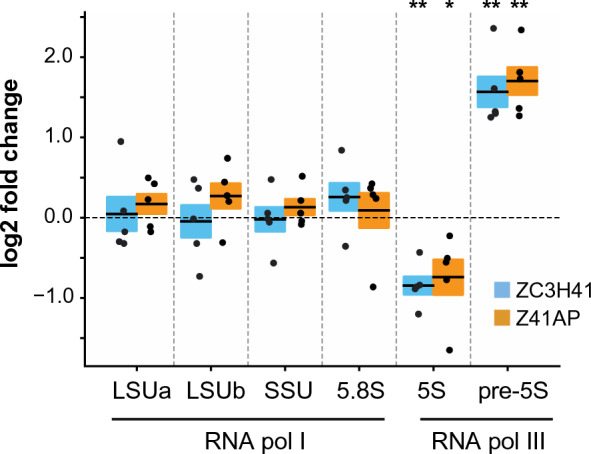
Effect of ZC3H41 or Z41AP depletion on the levels of different ribosomal RNAs (rRNAs). Total RNA was extracted from uninduced and ZC3H41- or Z41AP-depleted cells, and the levels of the indicated rRNA species (transcribed by RNA polymerase I or III, as indicated) were assayed by quantitative RT-PCR. Fold changes (log2 converted) relative to uninduced cells are expressed as the mean (horizontal solid lines) ± SEM (shaded areas) of five independent RNAi inductions (dots). The horizontal dashed line indicates log2 = 0, i.e. no change in gene expression. Two-sided Student’s *t*-tests (*n* = 5) were used to assess whether rRNA levels were significantly altered upon depletion of ZC3H41 or Z41AP. * P < 0.05, ** P < 0.005; differences in large subunit, small subunit and 5.8S levels were found to be not significant (*P* > 0.19 in all cases). For other abbreviations, see Figs. 1, 2 and 4

Several of the ZC3H41/Z41AP-associated mRNAs encode proteins that have been shown to be important for 5S rRNA maturation: NRBD2/p37 [10], RRS1 [11], and the ribosomal proteins L5 and L11 [12, 13] (Additional file 9). To investigate whether ZC3H41 and/or Z41AP have a role in this process, we measured the levels of mature 5S and pre-5S species by quantitative RT-PCR in ZC3H41- or Z41AP-depleted trypanosomes, and compared them to those of uninduced cells. As seen in Fig. 7, we could detect a significant > threefold (linear) increase in the levels of pre-5S upon knockdown of either ZC3H41 or Z41AP, whereas the amount of mature 5S rRNA significantly decreased about 1.7-fold in both cases. This effect seems to be specific for 5S rRNA, as the levels of mature large subunit, small subunit and 5.8S rRNA species did not significantly change in either RNAi. These results suggest that ZC3H41 and Z41AP are involved in the 5S rRNA maturation process in *T. brucei*.

**Fig. 8a, b:**
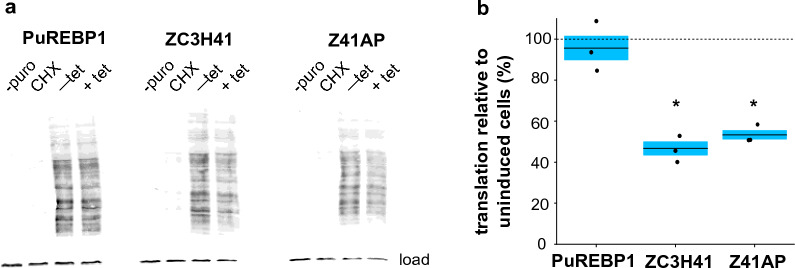
Effect of ZC3H41 or Z41AP depletion on protein synthesis.** a** Immunoblot showing puromycin incorporation into nascent polypeptides. Uninduced cells not treated with puromycin (− puro), uninduced cells incubated in the presence of cycloheximide 30 min prior to treatment with puromycin (CHX), uninduced cells treated with puromycin (− tet), cells treated with puromycin after 48 h of RNA induction (+ tet). Puromycilated polypeptides were detected using an anti-puromycin antibody. DRBD3 was used as a loading control. **b** Quantification of the puromycin signal from the immunoblot. Percentages of puromycilated polypeptides upon depletion by RNAi relative to those measured in uninduced cells are expressed as the mean (horizontal lines) ± SEM (shaded areas) of three independent RNAi inductions (dots). The horizontal dashed line indicates 100% puromycin signal relative to uninduced cells (i.e. no changes in translation). Two-sided Student’s *t*-tests (*n* = 3) were used to assess whether translation rates were significantly altered upon depletion of PuREBP1, ZC3H41 or Z41AP. * *P* < 0.05; differences observed upon depletion of PuREBP1 were found to be not significant (*P* = 0.56). For other abbreviations, see Figs. 1 and 2

The fact that the ZC3H41/Z41AP complex binds to ribosomal protein mRNAs and its loss seems to interfere with rRNA processing prompted us to assess whether ZC3H41 or Z41AP depletion had any effect on protein translation. To this end, we used the surface sensing of translation assay, which is based on the incorporation of puromycin into nascent proteins and subsequent detection of puromycilated polypeptides using an anti-puromycin antibody [52]. A significant twofold decrease in puromycin incorporation was observed upon depletion of ZC3H41 or Z41AP compared to uninduced cells (Fig. 8), indicating that loss of ZC3H41/Z41AP led to a global reduction in translation. These changes are in the same range as those reported for the RNA helicase Hel66, which is also involved in the maturation of rRNAs [8]. Importantly, translation was not significantly altered upon depletion of the RNA-binding protein PuREBP1 (Fig. 8), which is essential for cell growth [32], ruling out unspecific effects due to cell death.

## Discussion

Regulation of gene expression in trypanosomatids relies heavily on post-transcriptional mechanisms, and is exerted mainly through the action of RNA-binding proteins [18, 19]. Among these, CCCH-type zinc finger proteins have been shown to play important roles in the regulation of cell differentiation, protein translation, cell cycle and surface antigen expression [21–29]. ZC3H41 is a zinc finger protein that associates with stress and SL granules, and it is secreted in exosome vesicles [30, 31]; however, the function of this essential protein remains unclear to date. In this work we have shown that depletion of ZC3H41 results in severe cell cycle defects leading to a marked accumulation of zoids and multinucleated cells, suggesting that in the absence of ZC3H41, trypanosomes can progress through mitosis normally, but are then unable to segregate nuclei into daughter cells. ZC3H41 ablation also produced cells with an abnormal cell morphology elongation compatible with the nozzle phenotype observed for the first time in cells ectopically expressing the zinc finger protein ZFP2 [21], and later on upon ablation of the nuclear RNA-binding protein RRM1 [65], the cyclin CYC2 [66] and the ethanolamine-phosphate cytidylyltransferase [67]. It is still unknown what triggers the appearance of nozzled cells, but it could be due to mechanisms related to cell differentiation [17].

ZC3H41 forms a stable heterodimer with a protein that we have named Z41AP in this work. Both proteins are able to bind RNA as judged by UV crosslinking and PNK labeling experiments. Z41AP is also essential for proliferation in procyclic trypanosomes, but cell cycle and cell morphology were little affected in Z41AP-depleted trypanosomes, indicating that ZC3H41 could have additional and specific functions within the heterodimer. In agreement with this, ZC3H41 and Z41AP are apparently stable in the absence of the corresponding partner, and inhibition of cell growth was more evident in ZC3H41-depleted trypanosomes (i.e. compare Fig. 1a with Fig. 2c). Alternatively, these differences could be simply due to lower RNAi efficiencies in Z41AP-depleted cells. The ZC3H41/Z41AP heterodimer was also associated with both poly(A)-binding proteins, PABP1 and PABP2, and with the translation factors eIF4E4. It has been shown that PABP1 and PABP2 form distinct protein complexes in *T. brucei*, and that ZC3H41, Z41AP and eIF4E4 are found associated predominantly with PABP1 complexes together with the RNA-binding protein RBP23 and the translation factor eIF4G3; ZC3H41 and Z41AP also associate with PABP2 complexes, to a lesser extent [68]. Furthermore, PABP1, ZC3H41, Z41AP and eIF4E4 co-purify with the RNA-binding protein RBP23 in the related parasite *Leishmania infantum* [69]. Our TAP data show that PABP1, PABP2 and eIF4E4 are associated with the ZC3H41/Z41AP heterodimer in sub-stoichiometric amounts. Thus, although ZC3H41 and Z41AP seem to associate with PABP complexes (mainly PABP1 according to [68]), the ZC3H41/Z41AP heterodimer likely exists as a free species as well. This is supported by the observation that ZC3H41 and Z41AP are more abundant than PABP1, and are also in clear excess relative to either eIF4E4 or RBP23 proteins (Additional file 5). In addition, ZC3H41 and Z41AP could be present as independent entities as well. Ribosomal proteins were also detected in TAP-purified ZC3H41/Z41AP complexes. These probably represent contaminants, as ribosomal proteins are common in TAP purifications of other RNA-binding proteins [70]. However, a specific binding of ZC3H41/Z41AP to ribosomes is also plausible, since ZC3H41 has been found associated with polysomes in *T. brucei* [71].

Depletion of ZC3H41 led to minimal changes in the transcriptome, suggesting that the main role of this protein is unrelated to mRNA turnover. RIP-seq of ZC3H41/Z41AP complexes, on the other hand, revealed that they are mainly associated with ribosomal protein mRNAs, as 72 of the 104 top-ranked bound transcripts encode ribosomal proteins. Excluding paralogs, 46 of the 77 protein components of the *T. brucei* ribosome [63, 72] are encoded by ZC3H41/Z41AP-associated mRNAs (Additional file 9). This number is probably even higher, as several ribosomal protein transcripts have MACS2 scores just below the threshold, and we have shown binding of sub-threshold mRNAs to ZC3H41/Z41AP (Additional file 9). Depletion of ZC3H41 did not alter the levels of the ribosomal proteins S9 and P0, nor those of p37/NRBD2 (which is also a component of the ribosome [63]), which suggests that ZC3H41 does not regulate protein abundance either. In agreement with this, a genome-wide survey for post-transcriptional regulators in *T. brucei* showed that tethering of ZC3H41 or Z41AP to a reporter gene did not result in apparent changes in gene expression [73].

As mentioned above, ZC3H41 and Z41AP associate with PABP1 complexes together with the RNA-binding protein RBP23 and the translation factor eIF4E4 [68]. It has been proposed that the *T. brucei* PABP1 complex is specialized in the regulation of a small subgroup of mRNAs probably encoding ribosomal proteins [68], in line with the observation that ribosomal protein mRNAs are found associated with RBP23 in *L. infantum* [69] and eIF4E4 in *T. brucei* [74]. Moreover, it has been shown that both ribosomal protein mRNAs and PABP1 complexes are selectively excluded from stress granules upon starvation [68], and we have demonstrated here that ZC3H41/Z41AP complexes are significantly less associated with transcripts encoding ribosomal proteins upon nutritional stress. Since ZC3H41 and Z41AP locate to stress granules in starvation conditions [31], it is tempting to speculate that these proteins are involved in the selective transport of ribosomal protein mRNAs within the cell, and that ZC3H41/Z41AP ribonucleoprotein complexes are remodeled in response to environmental cues. In this regard, ZC3H41 has been shown to accumulate in secreted exosome vesicles that are thought to transmit stress signals for communication between parasites [30]. Phosphorylation of ZC3H41 is increased upon heat shock stress, as identified from mass spectrometry-based phosphoproteomic analysis [75]. Therefore, it is also possible that ZC3H41 and/or Z41AP are phosphorylated in response to starvation stress as well, resulting in differential binding to target mRNAs depending on nutrient availability.

The presence of transcripts encoding several factors involved in the maturation of 5S rRNA prompted us to investigate whether depletion of ZC3H41 or Z41AP had any effect on 5S maturation. We could detect both accumulation of 5S rRNA precursors and a significant decrease of mature 5S species, whereas the levels of other rRNA species remained apparently unaltered. 5S rRNA is transcribed by RNA pol III, whereas all other rRNAs are synthesized by RNA polymerase I. Interestingly, we have shown that two components of the RNA polymerase III complex, RPC4 and RPC11, are found in ZC3H41/Z41AP ribonucleoprotein complexes. Thus, in addition to having a role in regulating the fate of ribosomal protein mRNAs, the ZC3H41/Z41AP complex seems to be involved in the biogenesis of 5S rRNA. In agreement with this, a fraction of ZC3H41 seems to localize inside the nucleus [54]. Both types of RNAs (mRNAs encoding ribosomal proteins, and 5S rRNA) are prominently abundant within the cell. Therefore, one would expect ZC3H41 and Z41AP levels to be correspondingly high as well. Indeed, both proteins are among the top 5% most abundant proteins in procyclic trypanosomes (Additional file 5), and are also clearly the most abundant when compared to all CCCH zinc finger proteins in *T. brucei* (Additional files 5, 10).

Depletion of ZC3H41 led to few changes in the transcriptome, and the levels of three different proteins encoded by ZC3H41/Z41AP-associated mRNAs were not apparently altered either. Thus, the function of the ZC3H41/Z41AP complex is likely related to the transport of a specific cohort of transcripts in response to environmental cues. This is supported by the fact that association to target mRNAs is diminished upon nutritional stress, and that, as mentioned above, ZC3H41 is enriched in vesicles secreted to the extracellular medium. Since ribosomal protein mRNAs are specifically enriched in the ZC3H41/Z41AP-bound transcriptome, and this complex is also involved in 5S rRNA processing, it is tempting to speculate that ZC3H41 and Z41AP are involved in controlling the fate of the translation machinery in response to environmental cues. In this regard, we have shown that loss of either ZC3H41 or Z41AP leads to a reduction in translation. In addition, ZC3H41/Z41AP could play important roles in other stages of the *T. brucei* life cycle. For instance, it is known that the translation efficiencies of ribosomal components are diminished in metacyclic infective forms of *Trypanosoma brucei* and *Trypanosoma cruzi* [76, 77], whereas translation of trans-sialidase virulence factors is upregulated in metacyclic *T. cruzi* trypanosomes [76]. Interestingly, Z41AP and ZC3H41 protein levels are 2.2- to 2.4-fold less abundant in non-dividing metacyclic trypanosomes than in proliferative procyclic forms of *T. brucei* [77]. Further insights into the role of the ZC3H41/Z41AP complex will greatly benefit from the study of these essential proteins in other trypanosomal life forms.

## Conclusions

ZC3H41 is an essential zinc finger protein of the CCCH family which is known to locate to stress granules and exosome vesicles upon stress; however, the function of this intriguing protein in RNA metabolism remains unknown. In this work we show that ZC3H41 forms a stable heterodimer with another essential RNA-binding protein, Z41AP, and that the heterodimer also associates with the PABP1 complex. Using RNA-seq we show that ZC3H41 and Z41AP bind to mRNAs encoding ribosomal protein mRNAs and factors involved in 5S rRNA biogenesis. We also show that these transcripts are less associated with ZC3H41/Z41AP upon nutritional stress, and that depletion of ZC3H41 or Z41AP results in defects in 5S rRNA processing and a decrease in global translation. We propose that the ZC3H41/Z41AP complex is involved in the selective transport of ribosomal protein mRNAs within the cell, and that these ribonucleoprotein complexes are remodeled in response to environmental cues. Our work provides an interesting basis on which to further explore how trypanosomes link nutritional sensing with regulation of gene expression.

### Supplementary Information

**Additional file 1: Table S1. **Oligodeoxynucleotides used in this work.

**Additional file 2: Figure S1. **Localization of TAP-ZC3H41 and flow cytometry of ZC3H41-depleted cells.** a** Immunoblot of the cell lines expressing TAP-tagged versions of ZC3H41 and Tb927.7.7460 (Z41AP). **b** TAP-ZC3H41 localization was analyzed by immunofluorescence using an anti-protein A antiserum. **c** Representative flow cytometry plots are shown for uninduced or ZC3H41-depleted cell lines stained with propidium iodide.

**Additional file 3: Figure S2. **RNA interference of ZC3H41 using a different double-stranded RNA. Cells expressing TAP-ZC3H41 were transfected with a different tetracycline-inducible RNAi plasmid. **a** Growth curve of uninduced or RNAi-induced trypanosomes. **b** ZC3H41-depleted cells were mounted in mounting medium containing DAPI and analyzed for the appearance of zoids and 'nozzled cells'.

**Additional file 4: Table S2. **List of proteins associated with ZC3H41.

**Additional file 5: Figure S3. **Protein abundances of ZC3H41 and Z41AP. Data from deep proteome surveys carried out in bloodstream and procyclic trypanosomes were plotted according to protein intensity-based absolute quantification (iBAQ) values or protein abundance rank positions. **a** Components of the PABP1 complex. **b** *Trypanosoma brucei* zinc finger proteins of the CCCH class.

**Additional file 6: Table S3. **Effect of ZC3H41 depletion on the transcriptome of procyclic *Trypanosoma brucei.*

**Additional file 7: Table S4. **Raw read counts mapped to transcripts upon depletion of ZC3H41 in procyclic *Trypanosoma brucei*.

**Additional file 8: Figure S4. **Additional information related to ZC3H41/Z41AP RIP-seq. **a** Correlation analysis between the percentages of immunoprecipitated target mRNAs and MACS2 score or edgeR fold change values. **b** RIP followed by quantitative RT-PCR to confirm association of the indicated transcripts with ZC3H41/Z41AP. **c** Effect of ZC3H41 depletion on NRBD2, P0 and S9 protein levels.

**Additional file 9: Table S5. **Transcripts associated with the ZC3H41/Z41AP complex.

**Additional file 10:** **Table S6. **Protein abundances of zinc finger proteins of the CCCH family in *Trypanosoma brucei*.

## Data Availability

The RNA-seq datasets generated during the current study are available at the Gene Expression Omnibus repository (NCBI), https://www.ncbi.nlm.nih.gov/geo/query/acc.cgi?acc=GSE213007.
