# Supplementary material for: Role of the RNA-binding protein ZC3H41 in the regulation of ribosomal protein messenger RNAs in trypanosomes
Source: Parasit Vectors. 2023 Mar 31;16:118. doi: 10.1186/s13071-023-05728-x (PMC10064699; doi:10.1186/s13071-023-05728-x)
Supplement: Supplementary file 2 — Additional file 2: Figure S1. Localization of TAP-ZC3H41 and flow cytometry of ZC3H41-depleted cells. a Immunoblot of the cell lines expressing TAP-tagged versions of ZC3H41 and Tb927.7.7460 (Z41AP). b TAP-ZC3H41 localization was analyzed by immunofluorescence using an anti-protein A antiserum. c Representative flow cytometry plots are shown for uninduced or ZC3H41-depleted cell lines stained with propidium iodide. [file 13071_2023_5728_MOESM2_ESM.pdf]

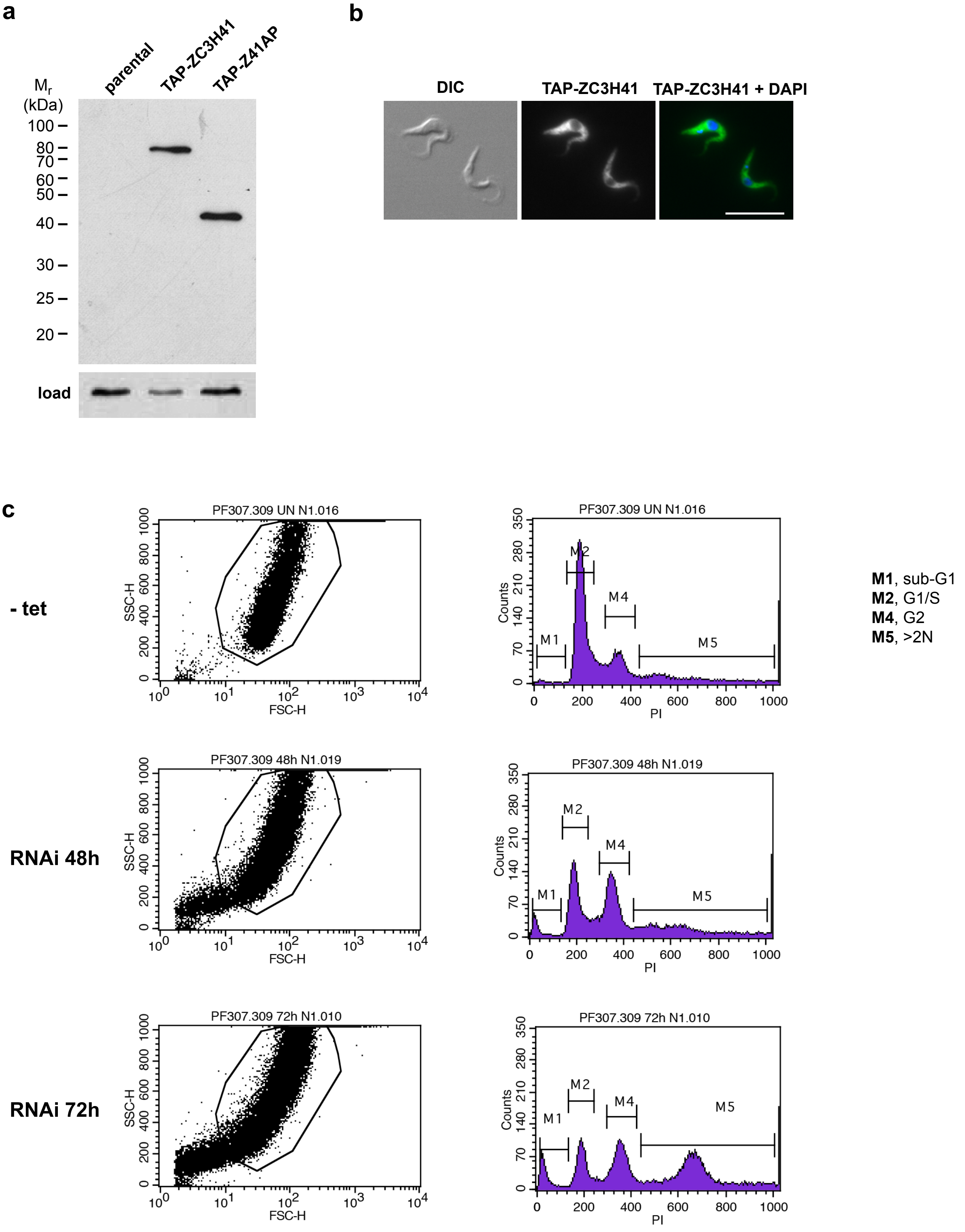

Additional file 2. Fig. S1 Localization of TAP-ZC3H41 and flow cytometry of ZC3H41-depleted cells. **a** Immunoblot of the cell lines expressing TAP-tagged versions of ZC3H41 and Tb927.7.7460 (Z41AP) used in this study. TAP-tagged proteins were detected using peroxidase anti-peroxidase (PAP) reagent. **b** TAP-ZC3H41 localization was analyzed by immunofluorescence using an anti-protein A antiserum, which binds specifically to the TAP tag. Bar, 10 µm. **c** Representative flow-cytometry plots are shown for uninduced or ZC3H41-depleted cell lines stained with propidium iodide. Density scatter plots representing complexity (SSC) and relative size (FSC) of a population of 20,000 cells are shown. Cells were gated (ovals) and histograms generated. M1, M2, M4 indicate sub-G1, G1/S and G2/M cells, respectively, whereas M5 refers to cells with more than two nuclei (>2N)
