# Supplementary material for: Role of the RNA-binding protein ZC3H41 in the regulation of ribosomal protein messenger RNAs in trypanosomes
Source: Parasit Vectors. 2023 Mar 31;16:118. doi: 10.1186/s13071-023-05728-x (PMC10064699; doi:10.1186/s13071-023-05728-x)
Supplement: Supplementary file 3 — Additional file 3: Figure S2. RNA interference of ZC3H41 using a different double-stranded RNA. Cells expressing TAP-ZC3H41 were transfected with a different tetracycline-inducible RNAi plasmid. a Growth curve of uninduced or RNAi-induced trypanosomes. b ZC3H41-depleted cells were mounted in mounting medium containing DAPI and analyzed for the appearance of zoids and 'nozzled cells'. [file 13071_2023_5728_MOESM3_ESM.pdf]

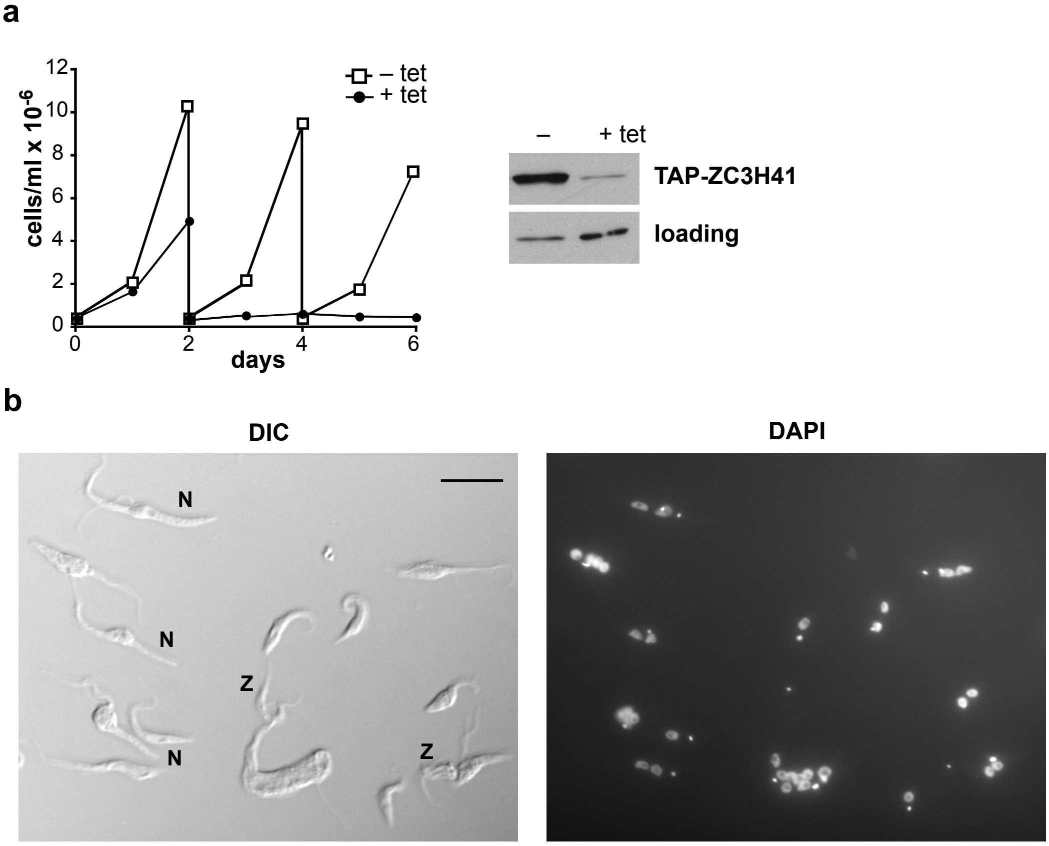

Additional file 3. Fig S2. RNA interference of ZC3H41 using a different dsRNA. Cells expressing TAP-ZC3H41 were transfected with a different tetracycline-inducible RNAi plasmid expressing a dsRNA corresponding to nucleotides 736 to 1101 of the ZC3H41 ORF; this fragment does not overlap with the original fragment used for ZC3H41 depletion. **a** Growth curve of uninduced or RNAi-induced trypanosomes, treated as described in Fig. S1 legend. Depletion of TAP-ZC3H41 was monitored by immunoblot after 48 h of tetracycline induction; DRBD3 served as a loading control. **b** ZC3H41-depleted cells were mounted in mounting medium containing DAPI and analyzed for the appearance of zoids (Z) and 'nozzled cells' (N). Bar, 10 μm.
