# Supplementary material for: Role of the RNA-binding protein ZC3H41 in the regulation of ribosomal protein messenger RNAs in trypanosomes
Source: Parasit Vectors. 2023 Mar 31;16:118. doi: 10.1186/s13071-023-05728-x (PMC10064699; doi:10.1186/s13071-023-05728-x)
Supplement: Supplementary file 5 — Additional file 5: Figure S3. Protein abundances of ZC3H41 and Z41AP. Data from deep proteome surveys carried out in bloodstream and procyclic trypanosomes were plotted according to protein intensity-based absolute quantification (iBAQ) values or protein abundance rank positions. a Components of the PABP1 complex. b Trypanosoma brucei zinc finger proteins of the CCCH class. [file 13071_2023_5728_MOESM5_ESM.pdf]

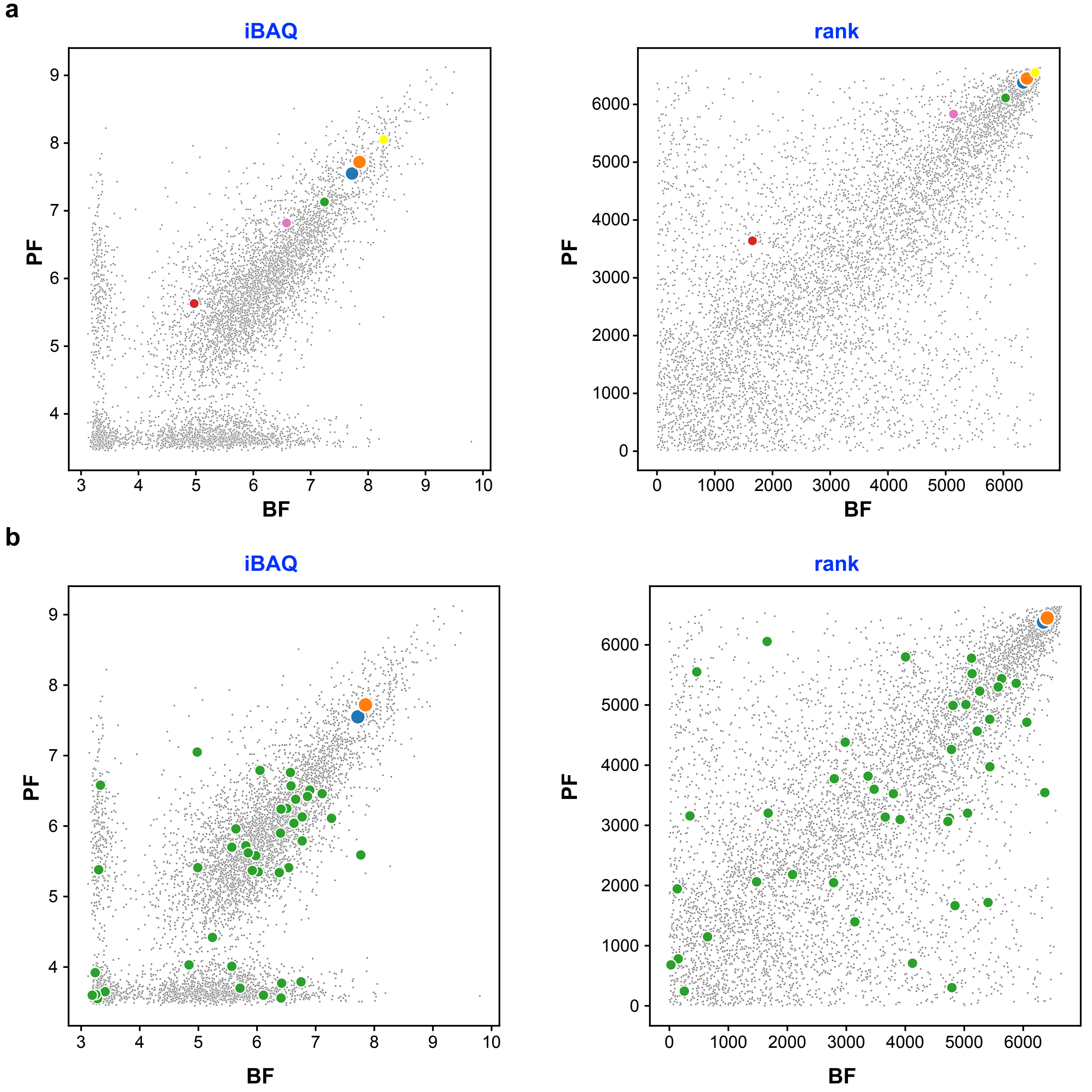

Additional fie 5. Fig. S3 Protein abundances of ZC3H41 and Z41AP. Data from deep proteome surveys carried out in bloodstream (BF) and procyclic (PF) strain 927 trypanosomes [56] were plotted according to protein iBAQ (intensity-based absolute quantification) values (left plots) or protein abundance rank positions from least (rank = 1) to most abundant (rank = 6643) according to iBAQ values (right plots). Each dot represents a protein. **a** Components of the PABP1 complex (PABP2 is shown as a reference): Z41AP (orange); ZC3H41 (blue); PABP1 (green); PABP2 (yellow); eIF4E4 (pink); RBP23 (red). **b** *T. brucei* Zn-finger proteins of the CCCH class are represented as green circles, except for ZC3H41 (blue). Z41AP (orange) is shown as a reference.
