# Supplementary material for: Role of the RNA-binding protein ZC3H41 in the regulation of ribosomal protein messenger RNAs in trypanosomes
Source: Parasit Vectors. 2023 Mar 31;16:118. doi: 10.1186/s13071-023-05728-x (PMC10064699; doi:10.1186/s13071-023-05728-x)
Supplement: Supplementary file 8 — Additional file 8: Figure S4. Additional information related to ZC3H41/Z41AP RIP-seq. a Correlation analysis between the percentages of immunoprecipitated target mRNAs and MACS2 score or edgeR fold change values. b RIP followed by quantitative RT-PCR to confirm association of the indicated transcripts with ZC3H41/Z41AP. c Effect of ZC3H41 depletion on NRBD2, P0 and S9 protein levels. [file 13071_2023_5728_MOESM8_ESM.pdf]

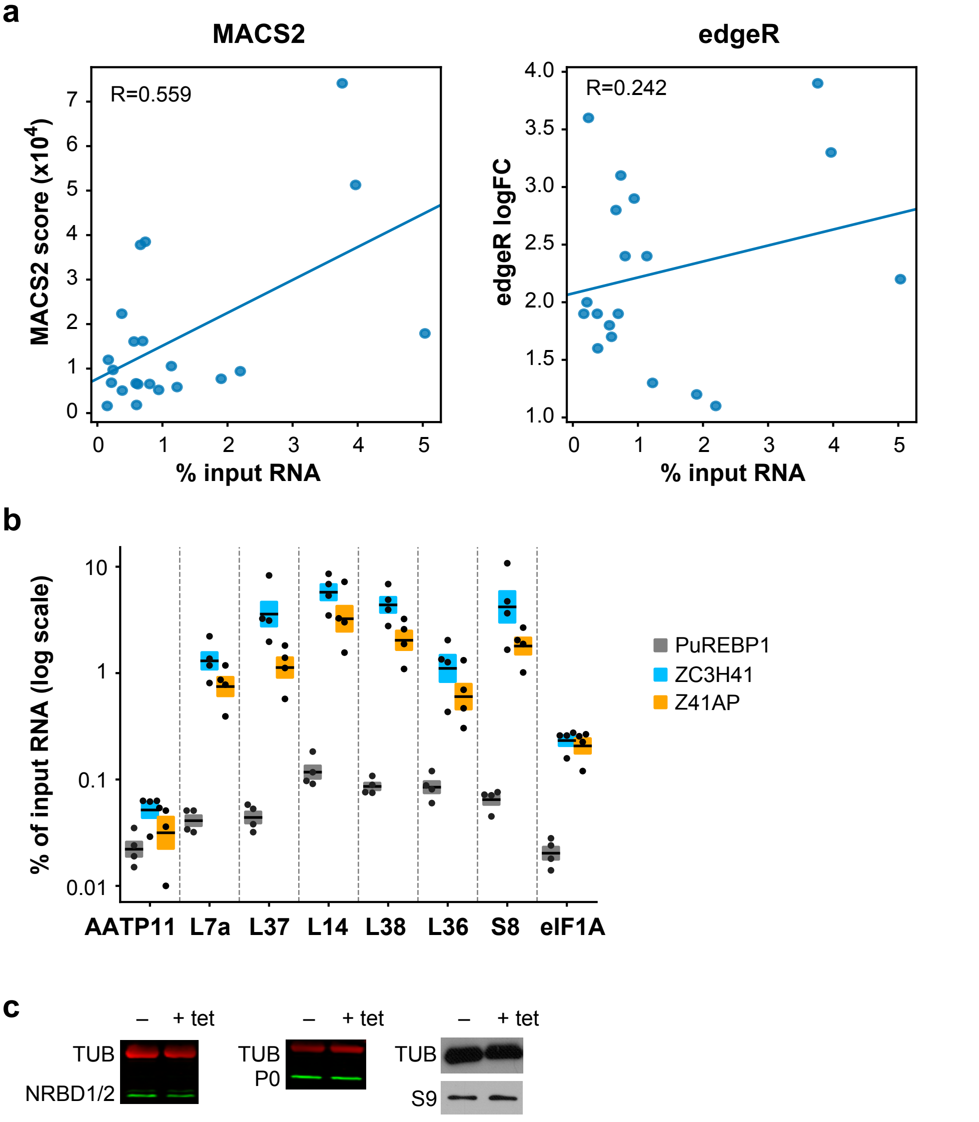


**Additional file 8. Fig S4.** Additional information related to ZC3H41/Z41AP RIP-seq. **a** Correlation analysis between the percentages of immunoprecipitated target mRNAs and MACS2 score (left) or edgeR fold-change values (right). **b** RIP followed by quantitative RT-PCR to confirm association of the indicated transcripts with ZC3H41/Z41AP; see Figure 5 legend for details. L38 data correspond to the Tb927.10.3280 paralog. **c** Effect of ZC3H41 depletion on NRBD2, P0 and S9 protein levels.
